# Neutrophil-to-Lymphocyte Ratio and Other Complete Blood Count Parameters in the Diagnosis of Serious Bacterial Infections in Febrile Infants Under Three Months

**DOI:** 10.7759/cureus.75945

**Published:** 2024-12-18

**Authors:** Aikaterini Mikelatou, Maria Eirini Gourtzelidou, Krino Maria Liveri, Athanasios Michos, Tania Siahanidou

**Affiliations:** 1 Pediatrics, First Department of Pediatrics, Medical School, National and Kapodistrian University of Athens, Athens, GRC; 2 Pediatrics, Postgraduate Program of Pediatric Infectious Diseases, Medical School, National and Kapodistrian University of Athens, Athens, GRC

**Keywords:** complete blood count (cbc), c-reactive protein (crp), febrile infant, inflammatory biomarker, lymphocyte-to-monocyte ratio (lmr), neonates, neutrophil-to-lymphocyte ratio (nlr), platelet-to-lymphocyte ratio (plr), serious bacterial infection, serious bacterial infection (sbi)

## Abstract

Background: The incidence of serious bacterial infections (SBI) in febrile infants under three months is high. Complete blood count parameters, an easily accessible and low-cost test, may have diagnostic potential for SBI.

Objectives: This study evaluated the efficacy of absolute neutrophil count (ANC), neutrophil-to-lymphocyte ratio (NLR), lymphocyte-to-monocyte ratio (LMR), platelet-to-lymphocyte ratio (PLR), platelet-to-mean platelet volume ratio (PLT/MPV), red cell distribution width (RDW), and C-reactive protein (CRP) in distinguishing febrile infants under three months with SBI.

Methods: A random sample of an eight-year registry-based retrospective cohort study included infants aged under three months who were hospitalized with fever. Patients were divided into those with and without SBI. Complete blood count parameters and CRP levels at admission, 24-48 hours, and discharge were compared between the groups.

Results: In total, 224 infants were included. At admission, infants with SBI (n=112) had higher median values (p=0.001) of CRP, ANC, and PLR and lower LMR (p=0.001) than those with negative cultures. CRP at admission was the best diagnostic marker for SBI (AUC: 0.857, 95% CI: 0.808-0.907), followed by ANC (AUC: 0.825, 95% CI: 0.772-0.879), NLR (AUC: 0.797, 95% CI: 0.738-0.855), and LMR (AUC: 0.642, 95% CI: 0.570-0.714). The combination of CRP with ANC, NLR, and LMR was found to be superior to any of the single markers (AUC: 0.918, 95% CI: 0.88 to 0.95). The combination of ANC and NLR proved to be a better discriminator compared to each parameter individually (AUC: 0.842, 95% CI: 0.79 to 0.90). At 24-48 hours, CRP continued to perform better, followed by ANC and NLR. At discharge, infants with SBI had higher PLT/MPV compared to those with negative cultures (p=0.009), but no significant differences in other parameters were observed.

Conclusions: The strongest predictor of SBI in febrile infants aged under three months was CRP, while ANC, NLR, and LMR also showed diagnostic potential. The combination of these parameters demonstrates the highest diagnostic value overall.

## Introduction

The occurrence of fever in infants under three months poses a significant challenge for pediatricians due to the high incidence of serious bacterial infections (SBI) in this age group. While fever is often attributed to self-limiting viral infections, 5% to 15% of these infants develop fever due to a SBI [1]. Diagnosing SBI in infants under three months is difficult because they lack typical signs and symptoms. The high rate of SBI and the risk of severe, potentially life-threatening complications make accurate diagnosis and immediate treatment crucial [2].

Efforts to create an evidence-based approach for evaluating and managing febrile infants under three months have been ongoing for over four decades. Despite the development of multiple criteria to stratify infants by SBI risk, no diagnostic approach has proven sufficiently sensitive and specific. As a result, clinicians often rely on invasive tests, such as suprapubic aspiration and lumbar puncture, and administer empirical broad-spectrum antibiotics, which extend hospitalization. However, these interventions carry potential adverse effects [1-3].

Since fever is often the only sign of illness in young infants, laboratory markers of infection are crucial for diagnosing infections, monitoring treatment response, and determining treatment duration. One widely studied marker is C-reactive protein (CRP). Although CRP is valuable in clinical practice, it rises only after several hours and lacks high specificity. There is growing interest in using procalcitonin as a biomarker of infection due to its promising ability to distinguish between bacterial and viral infections. However, this test is not widely available and is relatively expensive [3,4].

In the past decade, especially in the last four years, there has been increasing interest in the complete blood count (CBC) as an easily accessible and low-cost test for the diagnosis of various conditions. Numerous studies have aimed to highlight the combination of CBC parameters as prognostic and diagnostic indicators. Parameters such as absolute neutrophil count (ANC), neutrophil-to-lymphocyte ratio (NLR), lymphocyte-to-monocyte ratio (LMR), platelet-to-lymphocyte ratio (PLR), platelet-to-mean platelet volume ratio (PLT/MPV), and red cell distribution width (RDW) have shown statistically significant changes in conditions like cancer with poor prognosis, sepsis, and active autoimmune and rheumatic diseases [5-18].

However, only a few studies have investigated whether these parameters can distinguish febrile infants with SBI from those with benign, self-limiting illnesses. Therefore, our aim was to assess the potential of CBC parameters in identifying infants under three months of age with fever and SBI.

## Materials and methods

We conducted an eight-year, registry-based retrospective cohort study, including a random sample of infants under three months of age who were hospitalized with fever in the Neonatal Unit and Pediatric Clinics of “Aghia Sophia” Children’s Hospital between 2015 and 2023. The Ethical Committee of the First Department of Pediatrics of the National and Kapodistrian University of Athens approved the conduct of the study using material from hospital records (approval number: 1025/18.01.2022).

A total of 112 infants with SBI, defined as bacteremia, urinary tract infection (UTI), or meningitis, were randomly selected. Each infant with SBI was age-matched with a febrile patient without SBI, confirmed by negative culture results (no pathogen detected).

Data on the patient’s medical history and clinical presentation at admission were collected from their medical files. Complete blood count parameters, including ANC, NLR, LMR, PLT/MPV, PLR, and RDW, as well as serum CRP levels at admission, 24-48 hours, and before discharge, were recorded. Additionally, the results of cultures from biological fluids (blood, cerebrospinal fluid (CSF), and urine), the course of the disease, and patient outcomes were documented.

Patients with SBI, indicated by positive cultures of CSF, blood, or urine, were compared to those with negative cultures. Clinical findings at admission and laboratory parameters of the complete blood count at admission, 24-48 hours, and discharge were compared between the two groups. Finally, we evaluated the diagnostic efficacy of these parameters in differentiating febrile infants with SBI, determining the optimal cut-off values that exhibited the highest sensitivity and specificity.

Inclusion and exclusion criteria

Infants under three months of age with fever as the reason for admission, as well as those who underwent a complete blood count, serum CRP, blood culture, urine culture, and CSF cultures prior to the initiation of antibiotic treatment, were included in the study. Prematurity (gestational age <37 weeks), comorbidities (e.g., immunodeficiency or other chronic diseases), presence of a focal infection (e.g., soft tissue infection), and antibiotic therapy prior to admission were excluded from the study.

Definitions

Fever was defined as an axillary temperature measurement above 38°C [1-2]. SBI included bacteremia, UTI, and meningitis [19-20]. Bacteremia was defined as the growth of a single bacterial pathogen in the blood culture. The presence of skin-colonizing organisms that are not common human pathogens, such as coagulase-negative staphylococcus or viridans streptococci, in a single culture was considered sample contamination. Only their presence in multiple blood cultures was defined as a true infection [19].

UTI was confirmed by the growth of ≥100.000 colony-forming units (CFU)/mL in a urine culture from a clean-voided sample, by the growth of ≥10.000 CFU/mL of a single uropathogen in a catheter sample, or by the growth of ≥1.000 CFU/mL of a uropathogen in a suprapubic aspiration sample [20-21].

Meningitis was diagnosed by identifying a pathogenic microorganism in a CSF sample either by culturing the microorganism from the CSF or by detecting its genetic material using the polymerase chain reaction (PCR) test [18,19].

Statistical analysis

Data were analyzed using SPSS Statistics version 25 (IBM Corp. Released 2017. IBM SPSS Statistics for Windows, Version 25.0. Armonk, NY: IBM Corp.). A p-value of less than 0.05 was considered statistically significant. At baseline, categorical variables were summarized as counts and percentages, while continuous variables were presented as mean and standard deviation (SD) or median and interquartile range (IQR). The normality of continuous variables was assessed using Q-Q plots and histograms.

Comparisons between the two patient groups were made using the chi-square (χ²) test for independence and the Mann-Whitney test for quantitative characteristics. Each potential predictor underwent univariate analysis, with the results presented alongside 95% confidence intervals (CIs).

The multivariate logistic regression included the infection markers studied, and the probability calculated was the basis for the receiver operating characteristic (ROC) curve analysis. The discriminative ability of each studied predictor was observed using the area under the ROC curve (AUC), considering a high diagnostic value when the AUC exceeded 0.7. Cut-off values were determined using Youden’s index, calculated as max (sensitivity + specificity - 1). Additionally, the positive and negative predictive values of these tests were reported.

## Results

A total of 224 children were included in the study, with 112 of them diagnosed with SBI and 112 with negative cultures. The demographic characteristics of the study population are described in Table 1. The median age was 33.5 days (median = 38.7 days, IQR = 18.0-56.7 days), spanning from three to 90 days. Of the children included in the study, 121 (54%) were boys, and 103 (46%) were girls.

**Table 1:** Demographic characteristics of patients with SBI and with negative cultures SBI: serious bacterial infection

Variable subgroup	Ν (%) negative cultures (Ν=112)	Ν (%) SBI (Ν=112)
Sex		
Female	62 (55.4%)	41 (36.6%)
Male	50 (44.6%)	71 (63.4%)
Age		
≤28 days	47 (42.0%)	47 (42.0%)
29-60 days	43 (38.4%)	43 (38.4%)
>60 days	22 (19.6%)	22 (19.6%)

Evaluation at the emergency department

During the evaluation of infants in the emergency department (ED), fever height was recorded. Infants with SBI had significantly higher temperatures compared to those with negative cultures (median = 39°C vs. 38°C, p=0.001). Affected general appearance was more common in infants with SBI than in those with negative cultures (p=0.005). Additionally, a higher percentage of infants with SBI exhibited lethargy (p=0.022), grunting (p=0.005), and abnormal capillary refill time (p=0.02) compared to infants with negative cultures. Conversely, infants with negative cultures more frequently presented with symptoms such as cough (p=0.001), rhinorrhea (p=0.001), diarrhea (p=0.037), and vomiting (p=0.05). All these are detailed in Table 2.

**Table 2:** Clinical presentation at the ED and comparison between the two group of patients SBI: serious bacterial infection, ED: emergency department

Clinical presentation at ED	N (%), negative cultures (Ν=112)	N (%), SBI (Ν=112)	p-value
Affected general appearence	45 (40.2%)	66 (58.9%)	0.005
Lethargy	13 (11.6%)	26 (23.2%)	0.022
Grunting	19 (17.0%)	37 (33.0%)	0.005
Cough	52 (46.4%)	14 (12.5%)	0.001
Rhinorrhea	73 (65.2%)	32 (28.6%)	0.001
Diarrhea	18 (16.1%)	8 (7.1%)	0.037
Vomiting	20 (17.9%)	10 (8.9%)	0.050
Abnormal capillary refill time	20 (17.9%)	36 (32.1%)	0.020
Rush	16 (14.3%)	6 (5.4%)	0.051

Type of infection and detected pathogens

In a group of 112 infants with fever and SBI, 73 infants (65.2%) had UTI, 21 infants (18.8%) had UTI with accompanying bacteremia, 11 infants (9.8%) had bacteremia alone, three infants (2.7%) had both bacteremia and meningitis, three infants (2.7%) had meningitis alone, and one infant (0.9%) had both UTI and meningitis. Of the children with UTI, 70.6% were boys, while 29.4% were girls (p=0.002). The most frequently isolated pathogen in positive cultures was *Escherichia coli*, found in urine (86.3%), blood (40%), and CSF (28.5%). The detected pathogens are described in Table 3.

**Table 3:** Detected pathogens in urine, blood, and CSF cultures CSF: celebrospinal fluid

Pathogens	Urine cultures (N=95) N(%)	Blood cultures (N=35) N(%)	CSF cultures (N=7) N(%)
Escherichia coli	82 (86.3%)	14 (40%)	2 (28.5%)
Klebsiella pneumoniae	4 (4.2%)	4 (11.4%)	-
Enterococcus faecalis	3 (3.1%)	-	-
Klebsiella oxytoca	2 (2.1%)	1 (2.9%)	-
Enterococcus faecium	1 (1%)	1 (2.9%)	-
Enterobacter cloacae	1(1%)	2 (5.7%)	-
Salmonella enteritica	1 (1%)	2 (5.7%)	-
Streptococcus mitis	-	2 (5.7%)	-
Staphylococcus epidermidis	-	2 (5.7%)	-
Neisseria meningitidis	-	2 (5.7%)	2 (28.5%)
Streptococcus group C	-	1 (2.9%)	-
Streptococcus constellatus	-	1 (2.9%)	-
Listeria monocytogenes	-	1 (2.9%)	1 (14.3%)
Staphylococcus hominis	-	1 (2.9%)	-
Streptococcus agalactiae	-	1 (2.9%)	1 (14.3%)
Kluyvera intermedia	-	-	1 (14.3%)

For the febrile infants with negative cultures, a pathogenic virus was identified through rapid antigen tests or PCR in 57 infants (50.9%), classifying these cases as ''viral infections.'' The remaining 55 infants (49.1%) with negative cultures and no identified pathogenic strain were classified as having "unspecified infection." The type of infection and the detected pathogens are described in Table 4.

**Table 4:** Type of infection and pathogens detected at children with negative cultures RSV: respiratory syncytial virus, HHV6: human herpesvirus 6

Type of infection in children with negative cultures (N=112)	Detected pathogen	N (%)
Viral respiratory infection	-	31 (27.7%)
	RSV	15
	Rhinovirus	5
	Influenza type A	5
	Adenovirus	2
	Influenza type B	2
	Enterovirus	1
	HHV6	1
Viral meningitis	-	19 (17%)
	Enterovirus	13
	HHV6	3
	Human Parechovirus	2
	Adenovirus	1
Viral gastroenteritis	-	7 (6.3%)
	Enterovirus	3
	Adenovirus	2
	Rotavirus	2
Unspecified infection	-	55 (49.1%)

Comparison of laboratory parameters between the two groups of patients

At admission, infants with SBI had higher levels of CRP (p=0.001), ANC (p=0.001), and NLR (p=0.001) and lower levels of LMR (p=0.001) compared to those with negative cultures. No statistically significant differences were observed for PLR, PLT/MPV, and RDW between the two groups (Table 5). After 24-48 hours of hospitalization, infants with SBI continued to show higher levels of CRP (p=0.001), ANC (p=0.001), and NLR (p=0.001), and lower levels of LMR (p=0.038) compared to those with negative cultures. Again, no significant differences were found for PLR, PLT/MPV, and RDW (Table 6). At discharge, infants with SBI had higher PLT/MPV levels (p=0.009) compared to those with negative cultures, while no significant differences were detected for CRP, ANC, NLR, LMR, PLR, and RDW (Table 7).

**Table 5:** Complete blood count parameters and CRP in the two groups of patients at admission IQR: interquartile range, SBI: serious bacterial infection, CRP: C-reactive protein, RDW: red cell distribution width, ANC: absolute neutrophil count, NLR: neutrophil-to-lymphocyte ratio, LMR: lymphocyte-to-monocyte ratio, PLT/MPV: platelet-to-mean platelet volume ratio, PLR: platelet-to-lymphocyte ratio

Parameters at admission	Median (IQR) infants with negative cultures (Ν=112)	Median (IQR) infants with SBI (Ν=112)	p-value
CRP (mg/L)	4.0 (2.0-10.0)	33.0 (15.0-82.0)	0.001
RDW (%)	16.0 (15.0-17.0)	15.0 (15.0-17.0)	0.070
ANC (/μL)	3.832 (2.356-5.588)	7.354 (5.370-11.376)	0.001
NLR	0.83 (0.51-1.38)	1.79 (1.28-2.93)	0.001
LMR	3.47 (2.47-5.58)	2.71 (1.92-3.88)	0.001
PLT/MPV	46.590 (36.060-64.081)	50.900 (39.506-69.010)	0.286
PLR	95.45 (68.16-139.25)	106.06 (79.11-149.29)	0.114

**Table 6:** Complete blood count parameters and CRP in the two groups of patients at 24-48 hours of hospitalization IQR: interquartile range, SBI: serious bacterial infection, CRP: C-reactive protein, RDW: red cell distribution width, ANC: absolute neutrophil count, NLR: neutrophil-to-lymphocyte ratio, LMR: lymphocyte-to-monocyte ratio, PLT/MPV: platelet-to-mean platelet volume ratio, PLR: platelet-to-lymphocyte ratio

Parameters at 24-48 hours	Median (IQR) infants with negative cultures (Ν=112)	Median (IQR) infants with SBI (Ν=112)	p-value
CRP (mg/L)	3.0 (2.0-11.0)	34.0 (18.0-93.0)	0.001
RDW (%)	16.0 (15.0-17.0)	16.0 (15.0-17.0)	0.733
ANC (/μL)	3.530 (1.771-5.166)	6.429 (2.951-9.375)	0.001
NLR	0.68 (0.31-1.26)	1.23 (0.53-1.87)	0.001
LMR	4.74 (3.49-6.31)	3.79 (2.96-5.48)	0.038
PLT/MPV	46.606 (36.228-60.384)	52.545 (38.750-69.142)	0.116
PLR	91.95 (59.15-112.41)	91.50 (68.96-118.84)	0.303

**Table 7:** Complete blood count parameters and CRP in the two groups of patients at discharge IQR: interquartile range, SBI: serious bacterial infection, CRP: C-reactive protein, RDW: red cell distribution width, ANC: absolute neutrophil count, NLR: neutrophil-to-lymphocyte ratio, LMR: lymphocyte-to-monocyte ratio, PLT/MPV: platelet-to-mean platelet volume ratio, PLR: platelet-to-lymphocyte ratio

Parameters at discharge	Median (IQR) infants with negative cultures (Ν=112)	Median (IQR) infants with SBI (Ν=112)	p-value
CRP (mg/L)	2.0 (1.0-4.0)	2.0 (1.0-5.0)	0.378
RDW (%)	16.0 (15.0-17.0)	16.0 (15.0-17.0)	0.967
ANC (/μL)	2.247 (1.824-3.053)	2.885 (1.894-4.064)	0.057
NLR	0.38 (0.24-0.59)	0.44 (0.31-0.59)	0.200
LMR	6.06 (4.75-7.81)	6.55 (4.77-8.41)	0.308
PLT/MPV	53.924 (39.333-75.000)	64.593 (49.850-81.758)	0.009
PLR	79.94 (59.22-107.09)	88.00 (69.68-117.64)	0.076

​​​Diagnostic value of studied parameters

**Figure 1:**
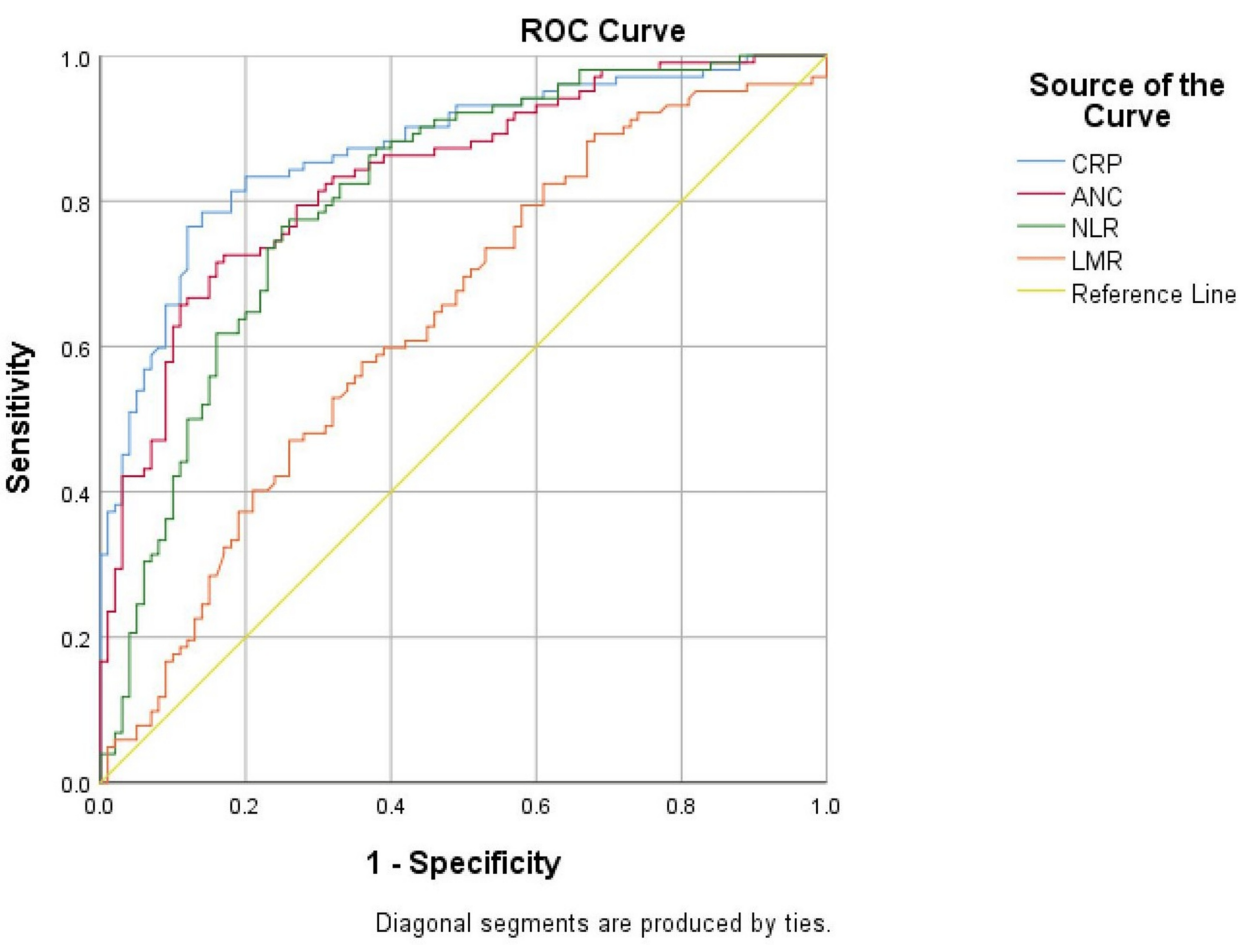
ROC curve for CRP, ANC, NLR, and LMR at admission ROC curve: receiver operating characteristic curve, CRP: C-reactive protein, ANC: absolute neutrophil count, NLR: neutrophil-to-lymphocyte ratio, LMR: lymphocyte-to-monocyte ratio

In the ROC analysis, CRP at admission proved to be the most effective diagnostic marker for SBI, with an AUC of 0.857 (95% CI: 0.808-0.907), followed by ANC with an AUC of 0.825 (95% CI: 0.772-0.879) and NLR with an AUC of 0.797 (95% CI: 0.738-0.855). LMR had a significantly lower diagnostic value, with an AUC of 0.642 (95% CI: 0.570-0.714) (Figure 1). The results, along with the cut-off values, estimated sensitivity, specificity, positive predictive value, and negative predictive value of the studied parameters, are detailed in Table 8.

**Table 8:** Diagnostic value of laboratory parameters on admission in distinguishing patients with SBI AUC: area under the curve, CI: confidence interval, PPV: positive predictive value, NPV: negative predictive value, CRP: C-reactive protein, ANC: absolute neutrophil count, NLR: neutrophil-to-lymphocyte ratio, LMR: lymphocyte-to-monocyte ratio

	AUC	95% CI	Cut-off value	Sensitivity	Specificity	PPV	NPV
CRP (mg/L)	0.857	0.808-0.907	10.9	81.3%	80.4%	78.2%	81.9%
ANC (μL)	0.825	0.772-0.879	6014	69.6%	83.0%	80.4%	73.2%
NLR	0.797	0.738-0.855	1.25	76.8%	71.4%	72.9%	75.5%
LMR	0.642	0.570-0.714	4.82	90.2%	33.9%	57.7%	77.6%

**Figure 2:**
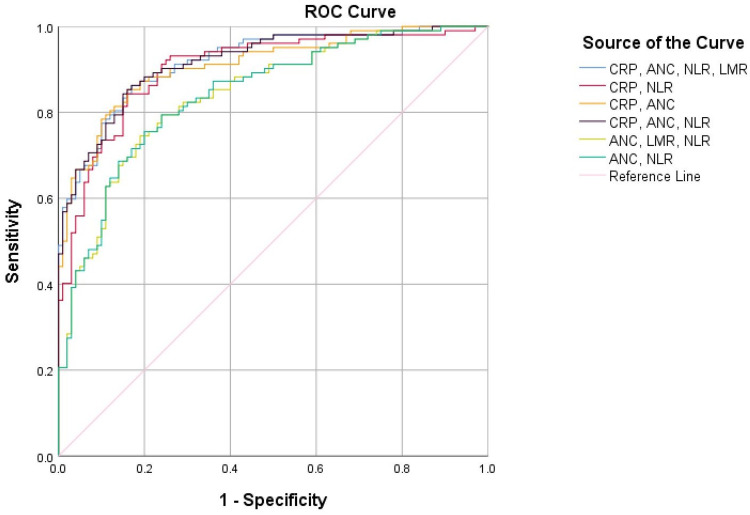
ROC curve for combined CRP, ANC, NLR, and LMR at admission ROC curve: receiver operating characteristic curve, CRP: C-reactive protein, ANC: absolute neutrophil count, NLR: neutrophil-to-lymphocyte ratio, LMR: lymphocyte-to-monocyte ratio

The combination of CRP with ANC, NLR, and LMR was found to be superior to any of the single markers with an AUC of 0.918 (95% CI: 0.88 to 0.95). A similar diagnostic value was presented from the combination of CRP with ANC and NLR with an AUC of 0.917 (95% CI: 0.88 to 0.95). Also, the combination of CRP with ANC and the combination of CRP with NLR showed a high diagnostic value with an AUC of 0.909 (95% CI: 0.87 to 0.95) and an AUC of 0.903 (95% CI: 0.86 to 0.95), respectively. Finally, the combination of ANC and NLR and the combination of ANC, NLR, and LMR were better discriminators than each individual studied parameter of the complete blood count with an AUC of 0.842 (95% CI: 0.79 to 0.90) and an AUC of 0.841 (95% CI: 0.79 to 0.89), respectively (Figure 2).

**Figure 3:**
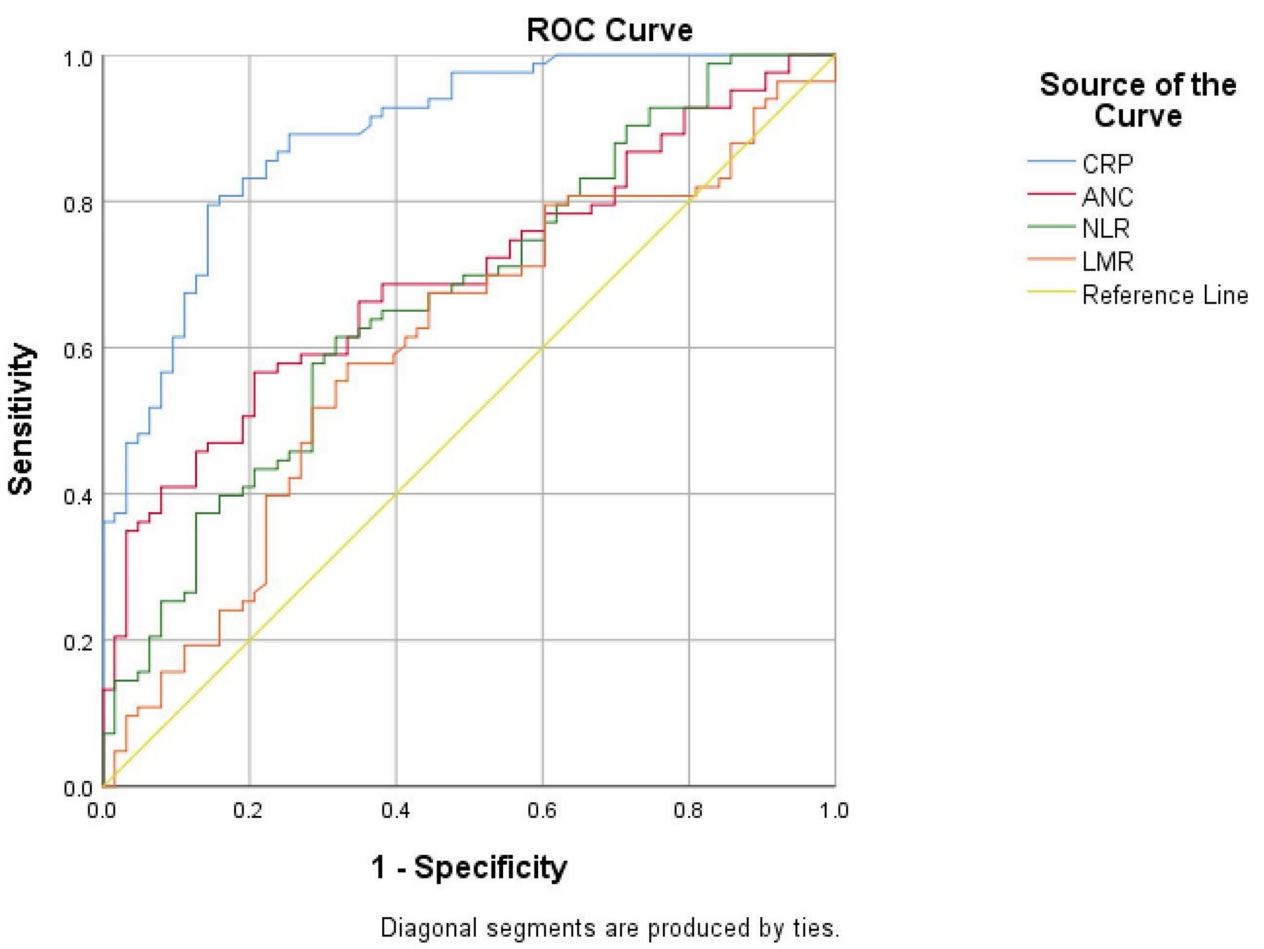
ROC curve for CRP, ANC, NLR, and LMR at 24-48 hours of hospitalization ROC curve: receiver operating characteristic curve, CRP: C-reactive protein, ANC: absolute neutrophil count, NLR: neutrophil-to-lymphocyte ratio, LMR: lymphocyte-to-monocyte ratio

At 24-48 hours of hospitalization, a high diagnostic value (AUC>0.7) in distinguishing febrile infants with SBI from febrile infants with negative cultures was observed for CRP (AUC: 0.892, CI: 0.84-0.94) and ANC (AUC: 0.702, CI: 0.62-0.78), while a statistically significant but lower diagnostic value was observed for NLR (AUC: 0.681, CI: 0.59-0.76) and LMR (AUC: 0.642, CI: 0.57-0.714) (Figure 3). Notably, CRP’s diagnostic value was higher at 24-48 hours of hospitalization compared to admission. Conversely, the complete blood count parameters (ANC, NLR, and LMR) at admission performed better than at 24-48 hours of hospitalization.

## Discussion

SBI in febrile infants, particularly those under three months old, are a major concern. Our study found that a significantly higher percentage of infants with SBI exhibited symptoms such as poor general appearance, lethargy, and grunting. In contrast, a higher percentage of infants with negative cultures showed symptoms like cough, rhinorrhea, diarrhea, or vomiting. Despite the above clinical symptoms and findings, young infants under three months often present with nonspecific symptoms, making it challenging to determine the source of infection. This highlights the importance of using decisive tests with quantitative measures to diagnose SBI in this age group.

In this study, we assessed the diagnostic value and the applicable thresholds of complete blood count parameters along with CRP in identifying SBI in febrile infants under 90 days old. Regarding the strengths and advantages of this study, it is among the few that included a “not healthy” control group. Additionally, by including lumbar puncture as an inclusion criterion, the study significantly reduces the possibility of central nervous system infection missing cases, thereby enhancing its reliability and accuracy. Notably, we recorded these parameters during the children’s hospitalization to better track their progress over time. Tracking these biomarkers longitudinally throughout the infection could provide valuable insights, with normalization potentially indicating recovery and aiding in decisions regarding the duration of antimicrobial treatment.

The results suggest that at the time of admission, CRP, ANC, and NLR were significantly higher (p<0.05) in febrile infants with SBI, whereas LMR was higher in infants with negative cultures. No significant differences were observed in PLR, PLT/MPV, and RDW between the two groups at admission.

CRP demonstrated the highest diagnostic value with an optimal cut-off of 10.9 mg/L, yielding a sensitivity of 81% and an NPV of 82%. The cut-off value identified in our study is lower than the ranges typically suggested in the literature (CRP >20 mg/L). However, lower cut-offs, such as 7.2 mg/L, have been reported for detecting SBI in infants under three months [4,7]. ANC followed with a high value and an optimal cut-off of 6.014/μL, showing a sensitivity of 70% and an NPV of 73%. In the literature, there are various optimal cut-offs for ANC. A threshold of 10.000/μL tends to increase specificity but decrease sensitivity. Lower cut-off values are often employed in combination with other criteria to balance this trade-off. For example, in PECARN analysis, an ANC of <4.090/μL, combined with a negative urinalysis result, achieved a sensitivity of 76.6%. The inclusion of procalcitonin (<1.71 ng/mL) was necessary to reach a high sensitivity of 97.7%, a specificity of 60.0%, and a negative predictive value of 99.6% [14].

In the initial hyperdynamic phase of infection, a proinflammatory state is established, driven by neutrophils, macrophages, and monocytes. Acute neutrophilia is driven by both enhanced production in the bone marrow and the release from the endothelial lining of blood vessels. This process is promoted by the production of endotoxin, tumor necrosis factor, interleukin IL-1, IL-8, and hematopoietic growth factors, such as granulocyte colony-stimulating factor. In the course of a systemic inflammatory response, neutrophil apoptosis is inhibited, and the adaptive immune response, driven by lymphocytes, is suppressed to prioritize the nonspecific immune response. Additionally, it is well established that severe infections such as sepsis can result in lymphocyte apoptosis and lymphopenia, which serves as an indicator of a worse prognosis [5-6,16]. Therefore, an increase in NLR and a decrease in LMR are anticipated during bacterial infections, particularly in severe infections.

In our study, NLR demonstrated a high diagnostic value at admission with an AUC of 0.797 (95% CI: 0.738-0.855) and an optimal cut-off of 1.25, yielding a sensitivity of 77% and an NPV of 76%. In Zhang et al.'s study, the AUC for NLR in distinguishing early neonatal sepsis was 0.788 (95% CI: 0.708-0.868) with a higher cut-off value of 3.1 (sensitivity: 77%, specificity: 78%) [13]. Hamiel et al. found that an NLR cut-off value of 2 showed a sensitivity of 52.3% and a specificity of 78% in identifying SBI in febrile infants under three months old [4]. Similarly, Chang et al. reported an AUC of 0.65 for NLR with a cut-off value of 1.24, which aligns closely with our study’s findings but with a significantly lower sensitivity of 57.4% and a specificity of 69.1% [17]. NLR is a valuable tool in the clinical assessment of febrile infants, particularly when used alongside other diagnostic markers [16,22].

LMR is known to decrease severe infectious diseases, autoimmune flare-ups, and cancer patients with poor prognosis [9,23]. To our knowledge, no research has been conducted on this indicator in febrile infants until now. In our study, LMR at admission was found to be significantly lower in infants with SBI, with an AUC of 0.642 (95% CI: 0.570-0.714) at a cut-off of 4.82, showing a sensitivity of 90.2% and a specificity of 33.9%.

Studies have shown that combining NLR with markers like CRP and ANC enhances the accuracy of diagnosing SBI [16,22]. In our study, the combination of CRP with ANC, NLR, and LMR was found to be superior to any of the single markers with an AUC of 0.918 (95% CI: 0.88 to 0.95). The combination of ANC and NLR was a better discriminator than the individual parameters, with an AUC of 0.842 (95% CI: 0.78 to 0.89).

RDW is a promising biomarker, according to the literature, particularly for identifying neonates with sepsis. RDW is higher in sepsis due to increased oxidative stress, which reduces the survival of RBCs and leads to the release of immature RBCs into the peripheral circulation. This results in greater variability in RBC size, thereby elevating RDW [14]. Nevertheless, in our study, RDW did not differ significantly between the two groups of patients. The absence of any diagnostic value of RDW in identifying SBI in our study population could possibly be attributed to the fact that we compared two groups of febrile infants, whereas, in most of the previous studies, neonates and infants with sepsis were compared to healthy controls. Consequently, these studies might have overestimated the significance of RDW as a diagnostic biomarker.

Reactive thrombocytosis, due to cytokine release in SBI, leads to a significant increase in platelet count. Platelet activation is induced by adenosine diphosphate, thromboxane A2, and platelet-activating factor and also by pro-inflammatory cytokines such as IL-1, IL-6, and tumor necrosis factor. A high platelet count indicates significant inflammation, while a low lymphocyte count suggests a poor immunological response to infection or a severe infection. Consequently, increased PLR levels are associated with severe systemic inflammatory conditions, such as sepsis [7]. However, in our study, no significant difference was observed in PLR between infants with SBI and those with negative cultures at admission, 24- 48 hours of hospitalization, or at discharge.

The number of platelets is typically inversely proportional to their average volume; as bone marrow production increases, platelet size decreases proportionally. Recently, PLT/MPV has been studied as a prognostic marker in critically ill adult patients with sepsis [22]. Specifically, a decrease in platelet count and an increase in mean platelet volume, resulting in a lower PLT/MPV ratio, have been associated with a worse prognosis. In our study, no significant difference was observed between the two groups of patients in PLT/MPV at the time of admission and 24-48 hours of hospitalization. However, before discharge, PLT/MPV was significantly higher in infants with SBI (p=0.009) than in infants with negative cultures.

At 24 to 48 hours of hospitalization, CRP demonstrated a higher diagnostic value compared to admission. Conversely, while ANC, NLR, and LMR could still distinguish infants with SBI from those with negative cultures, their diagnostic value was lower than at admission. This can be explained by the fact that neutrophils and monocytes, as part of the innate immune response, rise directly after the stimulus and gradually decrease thereafter (maximal response usually occurs within 4-24 hours). In contrast, CRP, an infection and inflammation marker, becomes detectable after six hours and peaks at 36-48 hours [4,5]. Despite their reduced diagnostic value, the decline in ANC and NLR and the rise of LMR can serve as indicators of therapeutic response within the first 24-48 hours, a period during which CRP is ineffective in this regard.

Regarding the study's limitations, it is important to note that the inclusion criterion of performing a lumbar puncture indicates that the majority of children in this study were in a compromised general condition. These children, under stress, may exhibit increased release of cytokines and catecholamines, leading to greater mobilization of neutrophils. This could have resulted in increased ANC and NLR values even in children with negative cultures within our sample, thus leading to an underestimation of the diagnostic ability of complete blood count parameters to distinguish SBI from infants with negative cultures. Despite multiple blood cultures being taken to identify potential pathogens in this group of patients, we cannot entirely rule out the possibility that they were misclassified into the culture-negative group. Furthermore, we excluded bacterial pneumonia and bone and soft tissue infections from our analysis of SBI. The prevalence of these conditions is not high in this population, and we did not anticipate their inclusion would significantly impact our results. Moreover, the diagnosis of these infections is likely easier due to the presence of focal inflammation and the availability of imaging tests for confirmation. Finally, it is important to highlight that, due to the low number of cases of meningitis or bacteremia, the analysis as a whole is more representative of UTI than of these conditions.

## Conclusions

In a population of febrile infants under three months of age, we found that CRP was the strongest independent predictor for SBI detection. However, CRP values are not always available. We showed that ANC, NLR, and LMR, which are readily available, can contribute to identifying children in the under-three-month age group who are at risk of SBI. The combination of CRP with these parameters showed a high diagnostic value, while the combination of ANC, NLR, and LMR performed better than they did individually. Including ANC, NLR, and LMR and their cut-off values in the automatic analyzers' complete blood count results can enhance patient management and support critical decision-making. Additionally, considering the increase in CRP within the first 48 hours, regardless of the application of antibiotic therapy, the parameters of the complete blood count can potentially serve as indicators of treatment response within this critical period. Overall, these biomarkers deserve increased recognition and further investigation in the context of infectious diseases.
